# Health-related quality of life after autologous stem cell transplantation for multiple myeloma or lymphoma impacts professional activity- an analysis from two German tertiary care centers

**DOI:** 10.3389/fonc.2026.1779331

**Published:** 2026-05-28

**Authors:** Anna Franziska Hamm, Ajelet Loesche, Christine Eisfeld, Evgenii Shumilov, Theo Leitner, Anne Letsch, Georg Lenz, Nikolas von Bubnoff, Daniela Vanessa Wenge, Cyrus Khandanpour

**Affiliations:** 1 Department of Hematology and Oncology, University Hospital Schleswig-Holstein, Luebeck, Germany; 2 Department of Medicine A, University Hospital Muenster, Muenster, Germany; 3 Department of Hematology and Oncology, University Hospital Schleswig-Holstein, Kiel, Germany; 4 Department of Pediatric Oncology, Dana-Farber Cancer Institute, Harvard Medical School, Boston, MA, United States; 5 Department Hematology, Hemostaseology, Oncology and Stem Cell Transplantation, Hannover Medical School, Hannover, Germany; 6 Department of Internal Medicine (Oncology and Hematology), University Medicine Oldenburg, Carl von Ossietzky University of Oldenburg, Oldenburg, Germany

**Keywords:** autologous stem cell transplantation, health related quality of life, multiple myeloma, outcomes, professional activity, return to work

## Abstract

**Purpose:**

Autologous stem cell transplantation (ASCT) for the treatment of multiple myeloma and lymphoma patients has been reported to improve relapse-free (RFS) and overall survival (OS). Nonetheless, the impact on patient-reported outcomes, such as professional activity and health-related quality of life (HRQoL) remains unclear.

**Patients and methods:**

In a cross-sectional two-center study, we collected data of 122 patients receiving high-dose chemotherapy followed by ASCT for multiple myeloma (MM) or lymphoma between 2007 and 2023 at two German tertiary care centers through standardized questionnaires.

**Results:**

The median age of all patients at diagnosis was 56 years (range 24–67 years). 17.9% of patients received a second ASCT. 55.0% and 61.9% of patients achieved a complete remission after first and second ASCT, respectively. The median time to resumption of employment was 167 days and the overall rate of return to work (RTW) was 56.0%. Following ASCT, 29.0% of patients reduced their working hours and 95.4% had a recognized disability status.

The median quality of life/health status QLQ-C30 score after ASCT was 66.7 out of 100. A QoL/health status of < 50 was significantly associated with a lower rate of resumption of professional activity (HR 0.44, 95% CI: 0.26-0.78, p = 0.02). 86.0% of all patients reported fatigue symptoms. The QLQ-MY20 score for fears of the future was significantly higher than for disease symptoms and therapy side effects (p < 0.0001), indicating that after ASCT, psychological symptoms affected patients more than somatic aspects.

**Conclusion:**

Our study suggests that long-term toxicities of ASCT significantly impact patients’ HRQoL and impair their professional activity. Treatment should include an assessment of mental health and socioeconomic status and provide necessary support.

## 1 Introduction

High-dose chemotherapy followed by autologous stem cell transplantation (ASCT) is a highly effective treatment for patients with malignant hematological neoplasia that has been used for decades (1).

The majority of patients currently receiving ASCT are patients with multiple myeloma (MM) (2). ASCT is still part of the first-line therapy for (transplant-)eligible MM patients, despite a wide range of recently developed targeted treatment options (1, 3–6). Moreover, ASCT is indicated for the treatment of patients with relapsed or refractory lymphomas (7) and solid tumors (8). ASCT and novel treatment agents have led to a significant increase in progression-free survival (PFS) and overall survival (OS) in MM patients (9–12). With improved treatment outcomes, patient-reported outcomes and health-related quality of life (HRQoL) assessment after ASCT have gained clinical significance and tools to investigate HRQoL and other patient-reported outcomes have been developed. They allow to incorporate multiple dimensions of patient function, including physical, emotional, social and symptom burden (13). The European Organization for Research and Treatment of Cancer (EORTC) Core Quality of life questionnaire (QLQ-C30) is one of the most frequently used tests in the literature and in practice to assess HRQoL, including 30 items and 15 scales (14).

High-dose chemotherapy followed by ASCT is commonly associated with 3–4 weeks of hospitalization (15). As acute mild to severe side effects are common, a decline in HRQoL is observed particularly in the early post-transplant period (16, 17). Previous studies suggest that full recovery is typically observed within the first two to six months following ASCT (16–18), but long-term side effects such as fatigue and depressive symptoms are not uncommon (16, 19, 20) and can affect patients’ mental health and financial security for years.

To date, comprehensive data on HRQoL of MM and lymphoma patients post ASCT and its impact on resumption of employment is scarce. Given that ASCT is often used in working-age patients, identifying its risks and long-term implications gains clinical importance and could allow for better patient counseling.

This study aims to shed light on patient-reported outcomes, such as subjectively perceived long-term toxicities and HRQoL as well as their impact on professional activity after ASCT in patients with MM and lymphoma.

## 2 Materials and methods

In this cross-sectional study, we included 122 patients receiving high-dose chemotherapy followed by ASCT for MM or lymphoma between 2007 and 2020 (University Hospital Muenster) and 2008-2023 (University Hospital Schleswig-Holstein, Campus Luebeck). Patients were contacted by telephone or directly in person during their appointments at the outpatient clinic. They answered the EORTC QLQ-C30 (21) and the multiple myeloma specific module Quality of Life Multiple Myeloma questionnaire (QLQ-MY20) (22). Answers were translated into scores from 0 to 100. These questionnaires were extended by sections on sociodemographic factors and professional activity. Patients completed the questionnaires at least 3 months after ASCT.

We retrospectively analyzed data by reviewing questionnaires and patient records. The Charlson Comorbidity Index (CCI) (23, 24) and the Eastern Cooperative Oncology Group (ECOG) (25) performance status were calculated using information from the patients’ medical record files. Analysis of patient-related data was approved by the local ethics committees (file number 2018-674-f-S, University Hospital Muenster; file number 2023-20, University Hospital Schleswig-Holstein, Campus Luebeck).

The primary endpoint analyzed in this study was HRQoL as determined by the EORTC-QLQ-C30 and QLQ-MY20 scores, assessing the overall quality of life/health status, role functions, treatment side effects and future perspectives of patients.

The secondary endpoint was the time to resumption of professional activity calculated from the day of ASCT. For patients who received more than one ASCT, the date of last ASCT was used. Cumulative incidence (CI) curves were used to estimate the RTW rate over time. Patients who were unemployed prior to diagnosis of MM or lymphoma were excluded from the calculation of CI.

Univariate analyses were performed using the log-rank-test for the cumulative incidence of resumption of employment and hazard ratios as well as 95% confidence intervals were calculated. For descriptive statistics, the two-tailed Mann Whitney test for two groups and the Kruskal-Wallis test with Dunn’s correction for multiple comparisons were utilized. Statistical analyses and data visualization were performed with GraphPad Prism 10 (Version 10.2.2).

## 3 Results

### 3.1 Patient characteristics

122 patients received high-dose chemotherapy with melphalan followed by ASCT for MM (n=102), amyloidosis (n=4) or high-dose chemotherapy with BEAM (carmustine (BCNU), etoposide, cytarabine and melphalan) for Hodgkin’s (n=4) or non-Hodgkin’s lymphoma (n=12) (**Table 1**).

**Table 1:** Disease and treatment characteristics of patients receiving high-dose chemotherapy with ASCT at both centers.

Disease characteristics	All patients n=122	Muenster n=70	Luebeck n=52
Disease subtype, n^1)^ (%): IgG myeloma IgA myeloma IgD myeloma light chain myeloma amyloidosis non myeloma^2)^	34/122 (27.9%) 49/122 (40.2%) 1/122 (1.8%) 18/122 (14.8%) 4/122 (3.3%) 16/122 (13.1%)	6/70 (8.6%) 46/70 (65.7%) - 14/70 (20.0%) 4/70 (5.7%) -	28/52 (53.8%) 3/52 (5.8%) 1/52 (1.9%) 4/52 (7.7%) - 16/52 (30.8%)
Gender, n (%): male female	72/122 (59.0%) 50/122 (41.0%)	42/70 (60.0%) 28/70 (40.0%)	30/52 (57.7%) 22/52 (42.3%)
Age at initial diagnosis (years), median (range) (n):	56 (19-67) (122)	57 (37-65) (70)	56 (19-67) (52)
ECOG^3)^, n (%): 0 1 2	35/106 (33.0%) 65/106 (61.3%) 6/106 (5.7%)	16/54 (29.6%) 33/54 (61.1%) 5/54 (9.3%)	19/52 (36.5%) 32/52 (61.5%) 1/52 (1.9%)
CCI^4)^, n (%): 2 3 4-6	62/122 (50.8%) 27/122 (22.1%) 32/122 (26.2%)	55/70 (78.6%) 8/70 (11.4%) 8/70 (11.4%)	7/52 (13.5%) 19/52 (36.5%) 26/52 (50.0%)
(revised) ISS at initial diagnosis, n (%): 1 2 3	32/83 (38.6%) 30/83 (36.1%) 21/83 (25.3%)	17/53 (32.1%) 21/53 (39.6%) 15/53 (28.3%)	15/30 (50.0%) 9/30 (30.0%) 6/30 (20.0%)
Osteolysis at initial diagnosis, n (%): yes	80/105 (76.2%)	49/70 (70.0%)	31/35 (88.5%)
Renal insufficiency (eGFR < 60 ml/min) at initial diagnosis, n (%): Yes	16/105 (15.2%)	12/70 (17.0%)	4/35 (11.4%)
Age at first auto SCT (years), median (range) (n):	57 (24-67) (122)	57 (38-66) (70)	60 (30-67) (52)
Complications during first auto SCT, n (%): Mucositis CTC 1-2 CTC 3 Infection CTC 1-2 CTC 3 Renal toxicity CTC 1-2 Bacteremia CTC 1-2 CTC 3	52/122 (42.6%) 21/122 (17.2%) 2/122 (1.6%) 40/122 (32.8%) 2/122 (1.6%) 4/102 (3.9%) 6/102 (5.9%)	28/70 (40.0%) 3/70 (4.3%) 2/70 (2.9%) 3/70 (4.3%) 1/70 (1.4%) 4/70 (5.7%) 1/70 (1.4%)	24/52 (46.2%) 18/52 (34.6%) - 37/52 (71.2%) 1/52 (1.9%) - 5/52 (9.6%)
Remission status after first ASCT, n (%): CR VGPR PR SD	60/109 (55.0%) 35/109 (32.1%) 11/109 (10.1%) 5/109 (4.6%)	24/57 (42.1%) 24/57 (42.1%) 10/57 (17.5%) 1/57 (1.8%)	36/52 (69.2%) 11/52 (21.2%) 1/52 (1.9%) 4/52 (7.7%)
Second ASCT, n (%): yes	21/122 (17.2%)	17/70 (24.3%)	4/52 (7.7%)
Complications during second ASCT, n (%): Mucositis CTC 1-2 CTC 3 Infection CTC 1-2 CTC 3 Renal toxicity	7/21 (33.3%) 3/21 (14.3%) 3/21 (14.3%) 2/21 (9.5%) -	4/17 (23.5%) 2/17 (11.8%) 1/17 (5.8%) 1/17 (5.8%) -	3/4 (75.0%) 1/4 (25.0%) 2/4 (50.0%) 1/4 (25.0%) -
Remission status after second ASCT, n (%): CR VGPR PR SD	13/20 (65.0%) 3/20 (15.0%) 2/20 (10.0%) 2/20 (10.0%)	10/16 (62.5%) 3/16 (18.8%) 2/16 (12.5%) 1/16 (6.3%)	3/4 (75.0%) - - 1/4 (25.0%)
Lenalidomide maintenance therapy post ASCT, n (%):^5)^ yes	49/100 (49.0%)	30/64 (46.8%)	19/36 (52.8%)

^1)^ General remark: “n” indicates the number of patients with data available for the respective category.

^2)^ Hodgkin’s lymphoma (n = 4), diffuse-large cell B-cell lymphoma (n = 5), follicular lymphoma (n = 2), mantle cell lymphoma (n = 2), T-cell lymphoma (n = 1), non-Hodgkin’s lymphoma, NOS (n = 1), T-cell and histiocyte-rich B-cell lymphoma (n = 1).

^3)^ Prior to ASCT.

^4)^ Post ASCT, at time of data acquisition.

ECOG, Eastern Cooperative Oncology Group status; CCI, Charlson comorbidity index; ISS, international staging system; eGFR, estimated glomerular filtration rate; CTC, common toxicity criteria; CR, complete remission; VGPR, very good partial response; PR, partial remission; SD, stable disease.

The median age of all patients at diagnosis was 56 years (range 19–67 years). At initial diagnosis, the (revised) International Staging System (ISS) score of MM patients was 1 (38.6%), 2 (36.1%) and 3 (25.3%), respectively. 75.5% of all patients presented with osteolysis at initial diagnosis and 15.1% were affected by renal insufficiency (**Table 1**).

Prior to ASCT, 33.0% of all patients presented with an ECOG status of 0, 67.0% had an ECOG status of 1 or 2 (**Table 1**). 50.8% of patients had a CCI of 2, 22.1% a CCI of 3 and 27.1% a CCI of > 3 (**Table 1**).

The median time between the first ASCT and completion of the QLQ-C30-MY20 questionnaire of all patients was 3.3 years (range 5.7 months - 15.0 years).

### 3.2 Treatment characteristics

The median age at first ASCT was 57 years (range 24-67) (**Table 1**). 17.2% of all patients received a second ASCT. The most frequent complication during first and second ASCT was mucositis (59.8% and 37.6%, respectively) followed by infections (34.4% and 23.8%, respectively). No liver toxicities and only mild renal toxicities in a few patients (CTC 1-2, 1.6% of patients) were observed during ASCT.

55.0% and 61.9% of patients achieved a complete remission after first and second ASCT, respectively (**Table 1**). 49.0% of patients with MM or amyloidosis received a maintenance therapy with lenalidomide (**Table 1**).

### 3.3 Health-related quality of life

**Figure 1:**
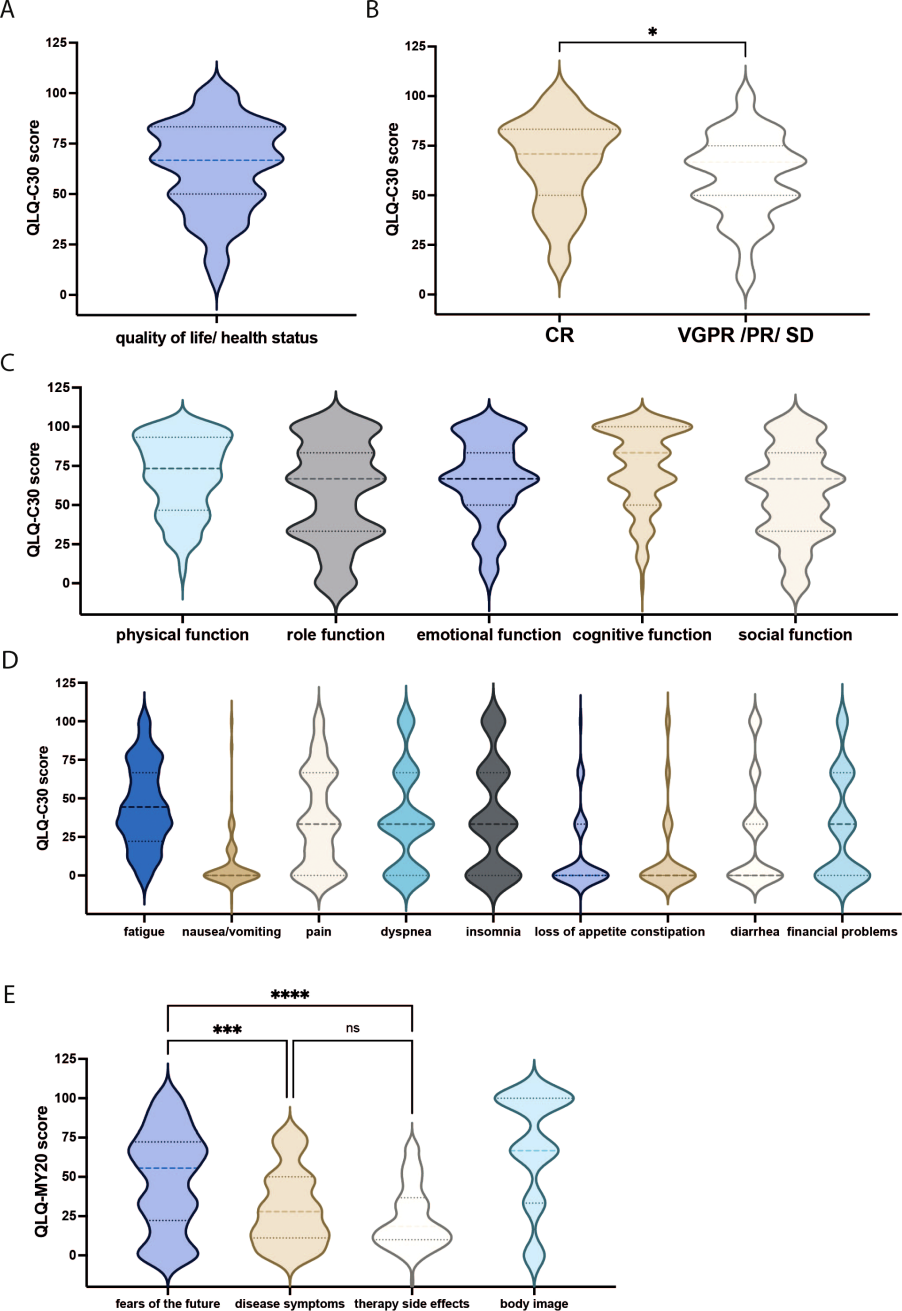
QLQ-C30 and QLQ-MY20 scores of all patients at the two centers. **(A)** Quality of life scores of all patients. **(B)** Quality of life/health status score based on remission status after ASCT. **(C)** QLQ-C30 scores for physical, role, emotional, cognitive and social function of all patients. **(D)** QLQ-C30 scores of somatic symptoms and financial problems of all patients. **(E)** QLQ-MY20 scores of all patients at the two centers. Median, 25^th^ and 75^th^ percentile indicated by dashed and dotted lines. CR, complete remission; VGPR, very good partial response; PR, partial response; SD, stable disease.

The median HRQoL/health status QLQ-C30 score of all patients after ASCT was 66.7, and only 5.7% of patients had a score of 100 (**Figure 1A**). Patients who achieved a complete remission (CR) after ASCT had a significantly higher HRQoL/health score than patients with very good partial response (VGPR), partial remission (PR) or stable disease (SD) (**Figure 1B**). Age, gender, the CCI or ECOG score as well as education level or remission status prior to ASCT did not significantly impact the QLQ-C30 quality of life/health score (**Supplementary Figure 1**).

The functional QLQ-C30 score was highest for cognitive function with a median score of 83, while the scores for role, emotional and social function were lowest (median score of 67) (**Figure 1C**).

Regarding the symptoms assessed by the QLQ-C30, fatigue impacted patients the most (median score of 44), with 86.0% of all patients reporting symptoms (**Figure 1D**). The second most common symptoms reported were pain, dyspnea and insomnia (median QLQ-C30 scores of 33.3, respectively). The least reported symptom was nausea and vomiting (median score of 0) (**Figure 1D**). Financial problems affected 47.2% of patients (median score 33.3) (**Figure 1D**).

Comparing the two centers, no significant differences between the QLQ-C30 scores for quality of life/health status, functions or symptoms could be detected (**Supplementary Figure 2**).

The QLQ-MY20 scores for fears of the future were significantly higher among all patients than the scores for disease symptoms and therapy side effects (*p* <.0001) (median scores of 55.6, 27.8 and 18.5, respectively) (**Figure 1E**). The highest QLQ-MY20 score among patients was for body image (median score 66.7) (**Figure 1E**). Comparing the QLQ-MY20 scores of the patients treated at the two different tertiary care centers, no significant differences were detected regarding fears of the future, disease symptoms or body image (data not shown).

### 3.4 Resumption of employment post auto SCT

**Figure 2:**
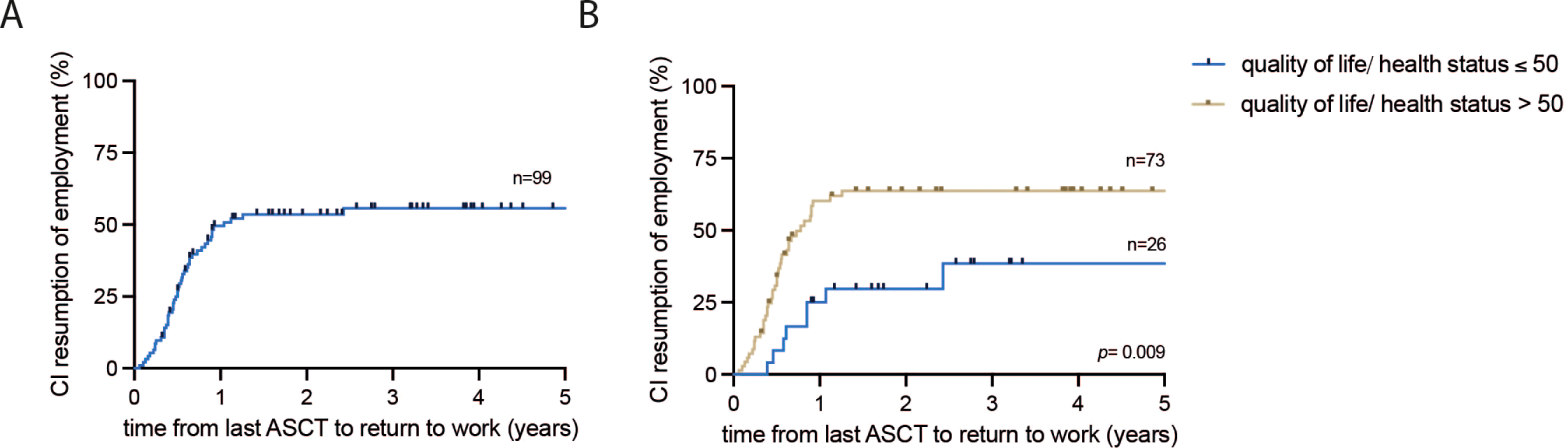
Cumulative incidence (CI) of resumption of employment post auto SCT.^1)^ **(A)** CI of return to work of all patients. **(B)** Probability of return to work stratified based on the quality of life/health status (QLQ-C30 score). ^1)^ Patients who were unemployed at the time of diagnosis were excluded from the analysis. auto SCT, autologous stem cell transplantation; CI, cumulative incidence.

82.0% of patients were employed prior to diagnosis of MM or lymphoma (**Table 2**). The CI of resumption of employment was 56.0% (**Figure 2A**) and the median time to RTW was 167 days. Following ASCT, 29.0% of patients reduced their working hours and 8.2% of patients changed their workspace (**Table 2**). 95.4% of all patients had a recognized disability status after ASCT (**Table 2**).

**Table 2:** Employment characteristics of patients after high-dose chemotherapy with ASCT at both centers.

Employment characteristics	All patients n=122	Muenster n=70	Luebeck n=52
Age at data acquisition (years), median (range) (n)^1)^:	61 (31- 71) (122)	61 (38-67) (70)	62 (31-71) (52)
Highest education level, n (%): training occupation A-levels/ university degree	73/105 (69.5%) 32/105 (30.5%)	43/62 (69.3%) 19/62 (30.6%)	30/43 (69.8%) 13/43 (30.2%)
Employed prior diagnosis, n (%): yes no	100/122 (82.0%) 22/122 (18.0%)	62/70 (88.6%) 8/70 (11.4%)	38/52 (73.1%) 14/52 (26.9%)
Employment prior to ASCT, n (%): employed self-employed retired	85/114 (74.6%) 14/114 (12.3%) 14/114 (12.3%)	53/62 (85.5%) 9/62 (14.5%) -	32/52 (61.5%) 5/52 (9.6%) 14/52 (26.9%)
Returned to work after ASCT, n (%): yes no	55/99 (55.6%) 44/99 (44.4%)	37/62 (69.7%) 25/62 (40.3%)	19/37 (50.0%) 19/37 (50.0%)
Employment post ASCT, n (%): employed self-employed unemployed, not searching for employment retired/ unable to work	45/99 (45.4%) 10/99 (10.1%) 3/99 (3.0%) 41/99 (41.4%)	33/62 (53.2%) 5/62 (8.1%) 3/62 (4.8%) 21/62 (33.3%)	12/37 (32.4%) 5/37 (13.5%) - 20/37 (54.1%)
Reduced working hours after ASCT, n (%): yes no	20/68 (29.4%) 48/68 (71.6%)	15/45 (33.3%) 30/45 (66.6%)	5/23 (21.7%) 19/23 (82.6%)
Changed workspace after ASCT, n (%)^4)^: yes no	5/60 (8.3%) 56/60 (91.7%)	5/37 (13.5%) 32/37 (86.5%)	- 23/23 (100.0%)
Disability status after ASCT, n (%): yes < 50% 50-70% >70-90% 100% no	104/108 (96.2%) 4/102 (3.9%) 47/102 (46.1%) 29/102 (28.4%) 22/102 (21.6%) 4/108 (4.6%)	59/62 (95.2%) 4/58 (6.9%) 24/58 (41.4%) 17/58 (29.3%) 13/58 (22.4%) 3/62 (4.8%)	45/46 (97.8%) - 23/44 (52.3%) 12/44 (56.8%) 9/44 (20.5%) 1/46 (2.2%)

^1)^ General remark: “n” indicates the number of patients with data available for the respective category.

A quality of life/health status QLQ-C30 score of > 50 was associated with a significantly higher cumulative incidence of RTW (*p* = .009) (**Figure 2B**). Also, the median time until resumption of employment was shorter in patients with a QLQ-C30 quality of life/health status > 50 (246 days vs not reached). No significant impact of gender, age, disease subtype, remission status prior or post ASCT, ECOG status, CCI score, the number of ASCT or education level on the CI of resumption of employment could be detected (data not shown).

In univariate analysis using the log-rank test, a quality of life/health status QLQ-C30 score of < 50 was associated with a significantly lower rate of resumption of professional activity (HR 0.44, 95% CI: 0.26-0.78, *p* = .02) (**Table 3**). Male patients showed a trend towards a higher RTW rate than female patients (HR 1.69, 95% CI: 0.99-2.86, *p* = .06). Also, patients with a better ECOG status (0 vs 1-3) and patients with a higher education level (A levels or university degree as highest degree vs training occupation) showed a trend towards a higher resumption of employment rate (HR 1.61 (0.86-3.00), *p* = .1 and HR 1.56 (0.88-2.78), *p* = .1, respectively) (**Table 3**).

**Table 3:** Univariate analysis of predictors of the resumption of employment for patients at both centers^1)^.

Predictors	n:	HR (95% CI):	p-value:
Quality of life/health status (QLQC-30 > 50/≤ 50)	73/26	2.65 (1.47-4.76)	**.009**
Gender (male/female)	56/43	1.69 (0.99-2.86)	.06
Age at diagnosis (< 60/60 years)	43/56	1.16 (0.68-1.96)	.59
ECOG status (0/1-3)	29/55	1.61 (0.86-3.00)	.10
CCI (2/3-5)	76/21	1.20 (0.64-2.25)	.54
Education (A levels or university degree/training occupation)	23/66	1.56 (0.88-2.78)	.10
Remission status prior first ASCT (CR/VGPR or PR or SD or PD)	20/80	1.08 (0.53-2.19)	.80
Remission status post first ASCT (CR/VGPR or PR or SD or PD)	52/37	1.40 (0.80-2.42)	.24
Number of ASCTs (1/> 1)	81/18	1.74 (0.90-3.35)	.17

^1)^ Refers to patients who were employed prior to diagnosis of myeloma or lymphoma. Bold values indicate statistical significance (p < 0.05).

## 4 Discussion

Here, we provide a detailed analysis of QoL and RTW of 122 patients who received ASCT for MM or lymphoma at two large tertiary care centers in Germany between 2007 and 2023.

This study focuses on the long-term side effects that can negatively impact the QoL for many years after ASCT and therefore have a major impact on the RTW rate of patients. Fatigue assessed by using the QLQ-C30 score, was reported by 86.0% of all patients in our cohort and represented the most frequent long-term side effect. In previous reports, MM patients had been reported to experience the greatest levels of fatigue and reduced QoL compared to patients with other hematological malignancies (26). The higher frequency of fatigue observed in our study may be explained by the relatively short median time between ASCT and survey in our cohort (3.3 years). However, previous reports comparing lymphoma patients treated with and without ASCT, did not show significant difference in the incidence of fatigue at 3 or 6 years after completion of therapy (18). This finding was confirmed in patients with Hodgkin’s lymphoma (20).

Furthermore, our data demonstrate that in addition to fatigue, functions of everyday life were impacted, as assessed by QLQC-C30 scores, with role, emotional and social function being most impaired.

Of note, in our cohort, the QLQ-MY-20 score for fears about the future was significantly higher than for somatic aspects such as disease symptoms and treatment side effects. This suggests that after ASCT, patients were more affected by psychological symptoms than by somatic aspects. As relapse of the primary disease and secondary malignancies are leading causes of long-term mortality after ASCT (56% and 25%, respectively) (2, 27), fears about the future as expressed by patients is a valid concern. It is reasonable to assume that patients who are in a superior remission status may have a higher level of confidence regarding the progression of their disease. That might explain the higher quality of life/health scores of patients in CR after ASCT as compared to those who achieved a VGPR, PR or SD. One limitation of the study is that the QLQ-MY20 score was used for all patients in this study, including the 13.1% of lymphoma patients so that lymphoma-specific issues such as B-symptoms or lymph node related symptoms were not specifically assessed.

Only few recent studies report on professional activity of patients after ASCT. In our study, 82.0% of patients were employed prior to diagnosis. The cumulative incidence of resumption of employment was 56.0% and the median time to RTW was 5.5 months.

The RTW rate observed in our cohort seems comparable to Scandinavian cohorts of lymphoma patients (RTW rate 50-58%) (28, 29) and higher than in previous reports of a European cohort of MM patients treated in the UK, Germany, Spain and Italy (RTW rate of 39.1%) (30, 31).

It is currently unclear which factors primarily influence resumption of employment after ASCT. Our study showed no significant correlation with gender, age, disease subtype, remission status prior or post ASCT, ECOG status, CCI score, number of ASCTs or education level. Some data published so far mentioned age >55 years, gender, number of children, family income or educational level as significant factors impacting the RTW rate (30, 32, 33). Among these, age appeared to be the strongest predictor for resumption of employment (30, 32, 33).

In our study, HRQoL/health status seems the most important determinant for the resumption of employment. Whether RTW leads to an improved perception of HRQoL/health status or whether a higher HRQoL/health status following ASCT caused a higher rate of RTW, cannot ultimately be concluded from our study. Potentially, both factors influence each other.

Apart from that, among patients resuming employment in our cohort, almost one third (29.0%) reduced their working hours after ASCT and 95.4% of patients had a disability status approved. Presumably, the patients’ ability to perform in their jobs is often impaired, even though they resume their employment.

A strength of our study is the simultaneous assessment of both health-related quality of life and return to work in a multicenter setting. Moreover, the combination of a universal with a disease-specific standardized questionnaire allows an in-depth analysis of long-term risks of ASCT in myeloma patients. Limitations of our study are the non-representative sample of patients and the missing information about drop-out patients. Therefore, selection bias cannot be ruled out. Another limitation is the relatively low number of lymphoma patients included in this study, impeding an analysis stratified by cancer entities. Further, the long period of study inclusion is a limitation, as HRQoL outcomes might have changed over time due to therapeutic advances. The results of this study can be applicable to countries with comparable social and health care systems. In countries with less financial support for patients on sick leave or with (occupational) disability status and without statutory pension insurance, the RTW rate might be higher due to financial problems despite of a lower HRQoL and higher symptom burden.

The results of this study can help to inform individual decision making of patients prior to ASCT, considering their personal situation including socioeconomic aspects.

Given that our study only contained 13.1% lymphoma patients, the findings mostly reflect outcomes for multiple myeloma patients. Further studies, including bigger cohorts of lymphoma patients, are needed to validate the findings of this study and to compare outcomes given differences in disease biology and time point of ASCT as recommended by clinical guidelines.

Our study suggests that long-term side effects after ASCT significantly impact patients’ QoL and impair their professional activity. As fatigue affects almost all patients following ASCT in our cohort, novel therapeutic options should be assessed regarding this relevant treatment side effect which seems to have a long-term impact on patient well-being. Psychological support should be offered to patients, given that after ASCT, psychological symptoms affected patients more than somatic aspects. Overall, treatment should therefore include an assessment of mental health and socioeconomic status and provide necessary support.

## Data Availability

The raw data supporting the conclusions of this article will be made available by the authors, without undue reservation.

## Supplementary material

Supplementary Figure 1 Quality of life analysis (QLQ-C30) depending on co-variates: **A.** age, **B.** gender, **C.** CCI, **D.** ECOG, **E.** education level, **F.** remission status prior auto SCT. Median, 25^th^ and 75^th^ percentile are indicated by dashed and dotted lines. CCI = Charlson Comorbidity Index, ECOG = Eastern Cooperative Oncology group, CR= complete remission, VGPR = very good partial response, SD = stable disease

Supplementary Figure 2 QLQ-MY20-scores of patients at the two centers Muenster and Luebeck. **A.** age, **B.** gender, **C.** CCI, **D.** ECOG, **E.** education level, **F.** remission status prior auto SCT. Median, 25^th^ and 75^th^ percentile are indicated by dashed and dotted lines.
