# Supplementary figures and images for: Health-related quality of life after autologous stem cell transplantation for multiple myeloma or lymphoma impacts professional activity- an analysis from two German tertiary care centers
Source: Front Oncol. 2026 May 28;16:1779331. doi: 10.3389/fonc.2026.1779331 (PMC13253377; doi:10.3389/fonc.2026.1779331)

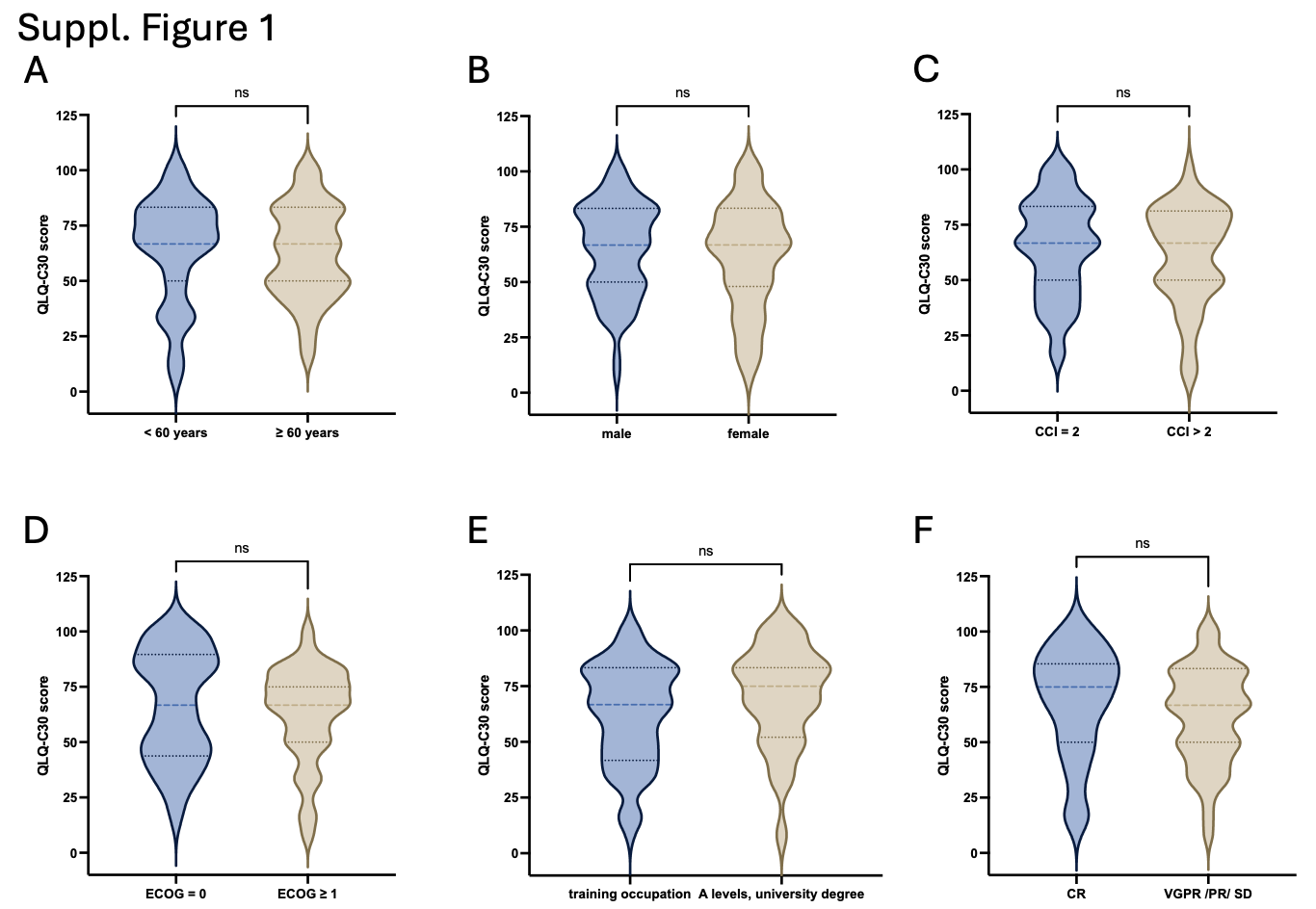

Supplement: Supplementary Figure 1 — Quality of life analysis (QLQ-C30) depending on co-variates: A. age, B. gender, C. CCI, D. ECOG, E. education level, F. remission status prior auto SCT. Median, 25th and 75th percentile are indicated by dashed and dotted lines. CCI = Charlson Comorbidity Index, ECOG = Eastern Cooperative Oncology group, CR= complete remission, VGPR = very good partial response, SD = stable disease [file Image1.png]

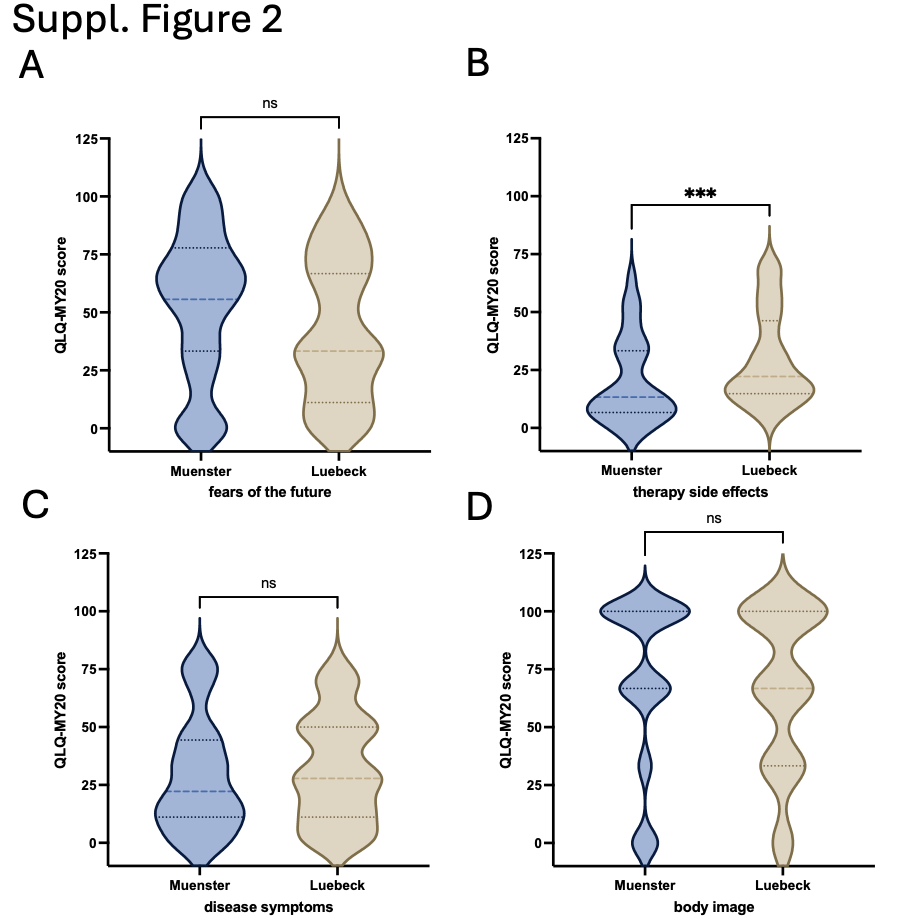

Supplement: Supplementary Figure 2 — QLQ-MY20-scores of patients at the two centers Muenster and Luebeck. A. age, B. gender, C. CCI, D. ECOG, E. education level, F. remission status prior auto SCT. Median, 25th and 75th percentile are indicated by dashed and dotted lines. [file Image2.png]
